# Institutional Needs

**Published:** 1915-12-11

**Authors:** 


### Institutional Needs.

INDUCED DRAUGHT PLANT FOR BOILERS.
(Messrs. Matthews and Yates, Ltd., Cyclone Works, Swinton, Manchester.)

Any system by which economy in fuel can be effected is especially worthy of consideration at the present time, when coal is so dear. In the Cyclone Induced Draught Plant Messrs. Matthews and Yates claim that its particular advantages lie in the fact that this plant increases the steaming capacity of the boilers to the maximum, and very often two boilers only [illegible] necessary where three have been previously used. Further, a cheaper quality and a less quantity of fuel may be used. By the use of this apparatus the black smoke nuisance is also obviated.

